# Retrieving ascarid and taeniid eggs from the biological remains of a Neolithic dog from the late 9th millennium BC in Western Iran

**DOI:** 10.1590/0074-02760160420

**Published:** 2017-09

**Authors:** Niloofar Paknezhad, Farbod Haji Mazdarani, Morteza Hessari, Iraj Mobedi, Faezeh Najafi, Negar Bizhani, Mahsasadat Makki, Gholamreza Hassanpour, Gholamreza Mowlavi

**Affiliations:** 1 Tehran University of Medical Sciences, Department of Parasitology and Mycology, School of Public Health, Tehran, Iran; 2 Islamic Azad University, Pre-historic Archaeology, Central Tehran Branch, Tehran, Iran; 3 University of Art, Faculty of Preservation and Restoration, Department of Archaeology, Isfahan, Iran; 4 Tehran University of Medical Sciences, Center for Research of Endemic Parasites of Iran, Tehran, Iran

**Keywords:** paleoparasitology, Neolithic time, dog, ascarid and taeniid eggs, Iran

## Abstract

**BACKGROUND:**

Paleoparasitology reveals the status of parasitic infections in humans and animals in ancient times based on parasitic particles found in biological remains from archaeological excavations. This line of research emerged in Iran in 2013.

**OBJECTIVE:**

The identification of parasites from Neolithic times is an attractive subject that shows the oldest origins of parasitic infections in a given geographical region. From an archaeological point of view, this archaeological site is well-known for animal domestication and agriculture in ancient Iran.

**METHODS:**

In this study, soil deposited on the surface and in the pores of a dog pelvic bone was carefully collected and rehydrated using trisodium phosphate solution.

**FINDINGS:**

The results showed ascarid and taeniid eggs retrieved from the biological remains of a dog excavated at the East Chia Sabz archaeological site, which dates back to the Neolithic period (8100 BC).

**MAIN CONCLUSION:**

The current findings clearly illustrate the natural circulation of nematode and cestode parasites among dogs at that time. These ancient helminth eggs can also be used to track the oldest parasitic infections in the Iranian plateau and contribute to the paleoparasitological documentation of the Fertile Crescent.

The identification of helminth eggs in ancient archaeological remains demonstrates the presence of human and animal parasitic infections over a long period of time in a particular geographical territory. The Neolithic period (9000-6000 BC) was when humans began animal domestication and agriculture, providing a driving force for the emergence of zoonotic parasites (Mazoyer & Roudart 2006). Animal parasites identified in this period would not be only considered of veterinary importance but may fill in the picture of human parasitic infection in the distant past. Parasitic fauna in humans would be expected to be transformed during Neolithic times as a consequence of such events (Bouchet et al. 2003). Although diverse parasite species existed on Earth before domestication and agriculture, the transmission of zoonotic infection to humans should have increased given the proximity and association between human and animals in this period (da Rocha et al. 2006). This issue has been documented by the retrieval of different helminthic eggs from archaeological sites around the world. Various helminths in carnivores, as well as *Fasciola hepatica* and *Dicrocoelium* sp. in herbivores in ancient Europe (Bouchet et al. 2003, Sianto et al. 2009), are documented examples of parasite transmission in this period. Intriguingly, the East Chia Sabz archaeological site located in Western Iran is among the world’s earliest and richest Neolithic sites with regard to its endowment of rich biological remains. Therefore, the discovery of ascarid and taeniid eggs from an excavated dog’s pelvic soil provides the focus for the current study, through which the emergence of some zoonotic helminths can be tracked in Iran and in the Middle East.

## MATERIALS AND METHODS

**Fig. 1:**
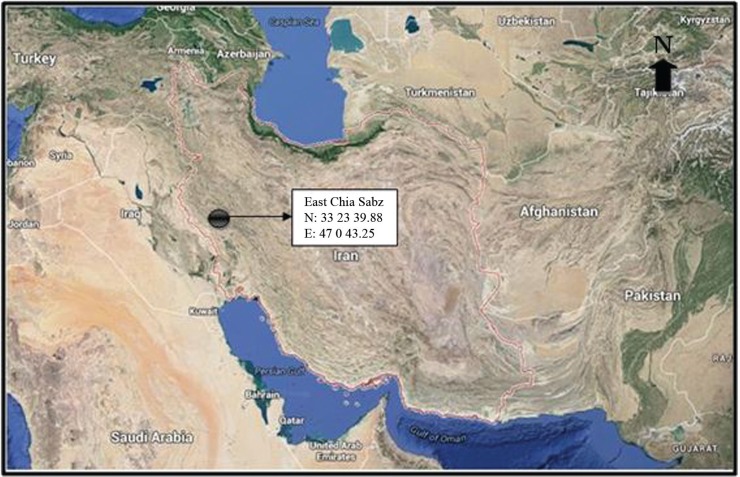
: Chia Sabz archaeological site location in Iran.

The East Chia Sabz archaeological site, located in Seymareh valley (Fig. 1), dates back to the Pre-Pottery Neolithic era (8100 BC) and provided the present paleoparasitological study materials. The soil deposited on the surface and in the pores of the pelvic bone of a dog were carefully collected and kept in plastic storage bags and transferred to the helminthology laboratory. A total of 7.5 g of the collected soil sample was rehydrated with a 0.50% (w/v) solution of trisodium phosphate (TSP) (Leles et al. 2010, Paknazhad et al. 2016). With this method, microscopic slides were permanently mounted using glycerin gel and were carefully studied for parasite eggs at different magnifications under a light microscope. The parameters of the eggs were measured and photographed using a microscope-equipped LABOMED Lx 500 camera. A morphological diagnosis of the eggs was carried out according to characteristics and morphometric parameters from reliable key references (Soulsby 1968, Meyers et al. 2000).

*Ethics* - The samples were provided in accordance with national legislation with no conflict of interest among the authors.

## RESULTS

**Fig. 2:**
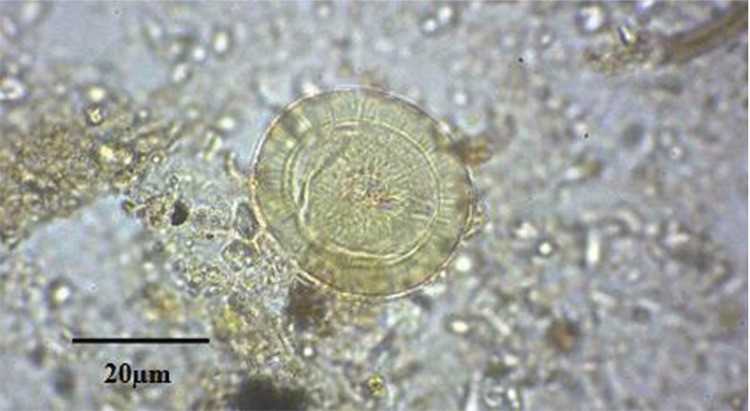
: taeniid egg found in soil deposited on the dog pelvic bone.

**Fig. 3:**
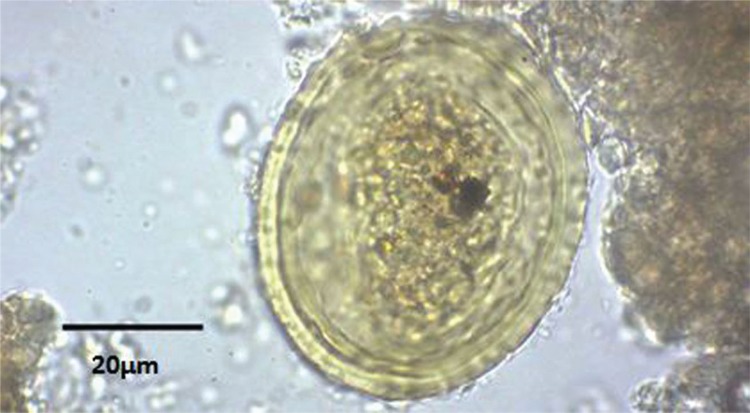
: ascarid egg retrieved from soil deposited on the dog pelvic bone.

Out of 50 grams of the soil collected from the dog’s pelvic bones, 7.5 gr was left submerged in the rehydration solution for 12 days. From the 126 slides studied, a *Taenia* sp. egg (Fig. 2) measuring 31.56 x 31.94 μm and an ascarid egg (Fig. 3) measuring approximately 63.42 × 51.55 µm with prominent spherical appearance were identified. On the latter egg, surficial pits were clearly visible.

## DISCUSSION

The Chia Sabz archaeological site has been widely acknowledged as the most important archaeological site in the Zagros mountainous area, with continuous human habitations and the presence of different biological remains (Mazdarani et al. 2014). The recovery of taeniid and ascarid eggs from the biological remains of a dog at this archaeological site is important not only from the point of view of antiquity but will also focus attention on the emergence of certain zoonotic helminths at that time in this part of the world. The finding of an ascarid egg with such morphological and size characteristics suggests an infection by a toxocarid nematode. Unfortunately, a deeper classification of the teaniid egg is not possible given the impossibility of differentiating tapeworm species from the family Taeniidae based only on egg morphology and size (Trachsel et al. 2007). Parallel to this understanding, the emergence of zoonotic infections such as visceral larval migrans as well as dog echinococcosis and human hydatidosis can be attributed to domestication (Higgs & Jarman 1969) and agriculture (Mazoyer & Roudart 2006), both having been practised since the Neolithic era. Worldwide reports of helminth eggs, primarily those common in humans and animals (Mitchell 2016), and an increase in human proximity with wild animals during this period (Dittmar 2009) support this idea epidemiologically. The domestication of dogs and goats, for instance, led to the occurrence of hydatidosis, toxocariasis, and fascioliasis among primitive populations in the Fertile Crescent (Diamond 2002). Although many different *Taenia* species are known to infect dogs, among which *T. hydatigena* and *T. multiceps* should be highlighted due to their frequency, one must consider the possibility that the taeniid egg found belongs to an *Echinococcus* species. Indeed, *E. granulosus*, which causes unilocular hydatidosis, is currently distributed in Mediterranean latitudes similar to those of our archaeological finding, and echinococcus-causing *E. multilocularis* is known to be a typical inhabitant of mountainous areas in Europe and is widespread in central Asia. In addition, this recovery of taeniid eggs in dogs reminds us of the potential existence of the most important tapeworm from a public health perspective, *Echinococcus* sp., in the study area.

Concerning the occurrence of visceral larva migrans in humans caused by ascarids in carnivores in ancient settlements, the proximity of humans and domesticated dogs should be regarded as a scenario similar to what has been described in human transmission patterns in areas with unfenced houses in the present (Núñez et al. 2013). The present ascarid egg with dimensions approximately 63.42 × 51.55 µm, slightly smaller than those of *T. canis* (90 × 75 µm) and *Toxascaris leonina* (75-85 × 60-75 µm) (Soulsby 1968), possibly due to taphonomic effects over an extended period of time, is indeed the oldest sign of human toxocariasis in ancient Mesopotamia. Nevertheless, the morphological appearance of the egg was typical enough to make a reliable diagnosis. The present paper describes the recovery of taeniid and most likely *T. canis* and/or *T. leonine* eggs in the biological remains of a Neolithic dog dating back to 8100 BC as the oldest paleoparasitological evidence in the Fertile Crescent.
